# The impact of pneumococcal conjugate vaccine on community-acquired pneumonia hospitalizations in children with comorbidity

**DOI:** 10.1007/s00431-016-2843-2

**Published:** 2017-01-09

**Authors:** Ellinor Sterky, Rutger Bennet, Ann Lindstrand, Margareta Eriksson, Anna Nilsson

**Affiliations:** 1 Department of Women’s and Children’s Health, Karolinska Institutet, Stockholm, Sweden; 2 Public Health Agency of Sweden, Solna, Sweden; 3 Paediatric Infectious Diseases Unit, Paediatric Emergency Department, Astrid Lindgren’s Children Hospital, Stockholm, Sweden; 4 Childhood Cancer Research Unit Q6:05, Astrid Lindgren Childrens Hospital, Stockholm, Sweden

**Keywords:** Childhood pneumonia, Pneumococcal vaccination, Hospitalization, Comorbidity

## Abstract

The burden of community-acquired pneumonia (CAP) in high-income countries is still significant. The introduction of pneumococcal conjugate vaccines (PCV) has reduced the overall need for hospitalization for CAP. However, it is not clear whether children with underlying disease also have benefitted from the PCV immunization programme. Children 0 to <5 years of age hospitalized with CAP and discharged with an ICD-10 code of J13-J18.9 between November 1, 2005, and April 30, 2007 (pre-vaccination period), and November 1, 2010, and April 30, 2012 (post-vaccination period), were eligible for this study. Data on hospitalization and discharge diagnoses were retrieved from the Hospital Registry. In addition, chart review was performed in 50% of the patients. Our result confirmed a decrease in hospitalization rate for CAP in the PCV13 period. Chart review revealed that half of the patients had underlying comorbidity and these children had more severe symptoms and required longer hospital stay. Intensive care was provided to less than 10% of the children and mostly for children with an underlying neurological disease.

*Conclusions*: We show that all children have benefitted from the reduction of CAP hospitalization after introduction of PCV. Our finding emphasizes the importance of children with chronic diseases receiving adequate vaccinations that may protect from lower respiratory diseases.

**Table:** 

**What is known?** • *Community-acquired pneumonia is a leading infectious cause of hospitalizations and death among children <5 years of age globally* • *Pneumococcal conjugate vaccine reduces the hospitalizations of all-cause pneumonia*
**What is new?** • *We show that also children with underlying comorbidities have benefitted from PCV immunization with a reduction of CAP hospitalization* • *We show that approximately half of all children hospitalized with CAP also have underlying comorbidities*.

## Introduction

Community-acquired pneumonia (CAP) is a leading infectious cause of hospitalizations and death among children <5 years of age globally [2, 13]. Among the estimated in-hospital deaths, the majority (99%) occur in low-income countries. Although the disease burden from childhood pneumonia is lower in high-income countries, medical service utilization, emergency visits and antibiotic use and related costs remain substantial. Despite this, there are relatively few studies related to the clinical burden in hospitalized children with pneumonia in high-income countries [12].

Major pathogens associated with childhood CAP include *Streptococcus pneumonia*, *Haemophilus influenzae*, *Staphylococcus aureus*, *Chlamydophila pneumoniae* and *Mycoplasma pneumoniae* [14, 21]. A number of viral pathogens including respiratory syncytial virus, rhinoviruses, human metapneumoviruses, adenoviruses and influenza have been acknowledged as important pathogens for CAP as well [8, 13]. However, identifying the cause of paediatric CAP has been problematic due to poor sensitivity of conventional microbial tests, such as blood cultures and nasopharyngeal (NP) cultures, to accurately identify bacteria involved in the localized lung infection. Molecular methods for identification of both viruses and bacteria may improve sensitivity, but perhaps not accurately predict viral vs. bacterial origin of CAP, since it has been increasingly recognised that healthy children may also be carriers of viruses in NP samples [16, 19]. The introduction of both *H. influenzae type b* and more recently pneumococcal conjugate vaccines (PCV) has had a great impact on CAP hospitalizations and mortality in both low- and high-income countries [5, 10, 11]. Pneumococcal conjugate vaccine PCV7, as the most studied vaccine so far, has repeatedly been reported to reduce hospitalizations due to pneumonia, which in most studies is defined as radiologically confirmed pneumonia, bacterial and non-bacterial pneumonia or cases identified through ICD codes. The range of reduction of all-cause pneumonia has been reported between 13 and 65% in countries as Italy, Poland, the US and Sweden after the introduction of PCV [1, 5, 10, 18].

However, there are still some unresolved problems related to the prevention and management of paediatric CAP in high-income countries. Hospitalization of children with CAP is already recommended for moderate-severe pneumonia, children with unstable vital parameters and in children with comorbidities. The most common comorbidities associated with CAP and/or invasive pneumococcal disease (IPD) are immunodeficiency, any type of childhood cancer, congenital heart or lung disease and neurological diseases [7, 8, 15]. Despite this, little data is available on the burden of CAP in children with comorbidities based on population data.

In Stockholm County, Sweden, PCV7 was offered on a 2 + 1 schedule at 3, 5 and 12 months of age to all children born since July 1, 2007. In January 2010, PCV7 was changed to PCV13 even for children who had received 1 or 2 doses of PCV7. No catch-up programme was implemented. High vaccine coverage was reached early on, and 98% of children born 2010 had received 3 doses of PCV [10]. Using a population-based retrospective cohort of children residing in Stockholm County, we sought to describe underlying comorbidities in children less than 5 years of age, hospitalized with CAP before and after the introduction of PCV. In addition, we provide data on the clinical course and complications for children with and without comorbidities.

## Material and methods

### Data source

Data for this retrospective cohort study were retrieved from the Hospital Registry, which contains information on hospitalization and discharge diagnoses covering all paediatric admissions in the Stockholm County. The three paediatric hospitals located in Stockholm participated in the study, all with paediatric emergency departments and one serving as a referral centre for ICU cases for children from the Stockholm County, Sweden. Incidence of pneumonia was calculated with population data from the Regional Planning Office, Stockholm County Council for the respective time periods. The population of children <18 years increased from 416,000 in the pre-vaccination period to 451,000 in the post-vaccination period. In addition, to validate diagnoses and retrieve in-depth data on comorbidity, 50% of all medical records were reviewed. The Regional Ethical Board in Stockholm, Sweden, approved of the study (D-no.: 2011/44-31/1).

### Patients

Children aged 0 to <5 years hospitalized for CAP were eligible for this study if they were residing in Stockholm county, discharged from the three participating hospitals between November 1, 2005 and April 30, 2007 (pre-vaccination period) and November 1, 2010 and April 30, 2012 (post-vaccination period). Subjects living in the Stockholm area were included if they were discharged with an ICD-10 code of J13-J18.9 which excludes a diagnosis of pneumonia of known viral origin. A readmission occurring within 30 days from first admission/discharge was checked through chart revision and excluded if considered a deterioration of the first episode. In the post-vaccination period, the vaccine up-take was estimated to 80% in our cohort based on the age of the child at admission in relation to the three-dose vaccine schedule. In children aged 2 to >5 years, 34 children (5.7%) were born before July 1 2007 and thus not offered PCV7.

### Study definitions

Patients with chronic comorbid conditions were identified through their respective discharge diagnosis and through chart review. The underlying diseases within each risk group in this study are shown in Table 1. During chart review, the CAP cases were further classified as severe using a previously published classification scheme [4]. For the pneumonia to be classified as severe, the child had to have ≥1 major criteria; need of mechanical ventilation, acute need of non-invasive positive pressure ventilation, hypoxemia (less than SpO2 below 90%) or >2 minor criteria; tachypnea greater than age-adjusted respiratory rates, apnoea, increased breathing work, altered mental status or unexplained metabolic acidosis [4].

**Table 1:** Definitions of chronic comorbidity used in this study and their prevalence in the chart review (*n* = 621) conducted from the two time periods

Risk group	Definition	Pre-vaccination (*n* = 180)	Post-vaccination (*n* = 162)
Obstructive disease	Children with asthma or recurrent obstructive bronchitis	85	94
Chronic lung disease	Children with bronchopulmonary dysplasia	8	8
Neurological disease	Children with impaired muscular tonus, cerebral palsy or severe epilepsy	21	28
Chronic heart disease	Children with congenital heart defects	3	0
Congenital malformations and/or syndromes	Children with craniofacial malformations, lung malformations, esofageal artresia	16	6
Other diseases^a^	Children with diabetes, cancer and primary immunodeficiency	47	26

^a^Chronic morbidities accounting for less than 5% in the cohort were grouped together as ‘other risk factorsʼ

### Statistics

Statistical analysis was done in SPSS 21.0 and Stata/IC 12.0. Fischer’s exact test and Chi square statistics were used to compare parameters in the pre- and post-period and to compare parameters between the children with and without risk factors. We stratified the subjects in two age groups (0 < 2 years of age, 2 to <5 years of age). Mann-Whitney tests were performed to compare median and mean values. A *p* value of <0.05 was considered significant.

## Results

### Cohort characteristics and total incidence of CAP hospitalizations

Patients hospitalized due to CAP are described in Table 2 for the two study periods. As expected, children <2 years of age accounted for approximately 60% of the hospitalized cohort in both time periods. The incidence of hospitalizations with pneumonia coded as bacterial was compared for the two periods. In children <2 years of age, the yearly incidence decreased from 5.1 (95% CI: 4.6–5.5) to 3.4 (3.1–3.7)/1000 children. In the older age group of 2- to <5-year-old children, a minor reduction was noticed from 2.5 (2.3–2.8)/1000 to 2.0 (1.8–2.3)/1000 children.

**Table 2:** Patient characteristic in the total cohort (*n* = 1332) based on ICD discharge diagnosis

	Pre-period	Post-period
Total cohort	739	593
Gender M (%)	350 (43)	329 (55)
Age group <2 years (% )	438 (59)	337 (57)
Age group 2 to <5 years (%)	301 (41)	256 (43)
Children with comorbidities (%)	210 (28)	197 (33)

### Severity of pneumonia, complications and mortality in the selected study population

For further understanding of the importance of comorbidities for hospitalization of children with CAP, patients (*n* = 621) were randomly selected for chart review (Table 3). This cohort was similar with respect to gender and age groups compared to all hospitalized CAP children (*n* = 1332), but the proportion of children with comorbidities was higher in the patient cohort where chart review was performed compared to data based on discharge diagnosis only (50 vs. 30%). Clinical parameters indicating severity of pneumonia were compared in children with or without risk factors (Table 3) for each study period. Children with comorbidity were classified with more severe pneumonia and were also cared for in the PICU more often. PICU care was in the majority of cases provided for children <2 years of age (data not shown). Complications to CAP were rare, but there were two children in the pre-immunization period and 6 children in the post-immunization period that suffered from a complication represented by 5 cases of empyema, one case of lung abscess, necrotizing pneumonia and septicaemia, respectively. In five children <2 years of age with severe underlying neurological disease, CAP was contributing to in-hospital death as revealed by chart review. Two of these children were born before the PCV introduction and where thus not offered PCV vaccination. Interestingly, when comparing the study cohort over the two periods, there were significantly more cases classified as severe pneumonia in the post-period (*p* = 0.035) (data not shown). We also recorded the length-of-stay (LOS) for the two study periods as it may correlate to severity and underlying comorbidity. In the pre-period, the median LOS was 2 days for all children under the age of 5, and this increased to 3 days in the post-period (*p* = 0.027). Children with comorbidities stayed significantly longer in the hospital than did children without previous illnesses (*p* = 0.000) during the whole period, including pre- and post-period (data not shown).

**Table 3:** Hospitalization data extracted by chart review (*n* = 621) from the pre- and post-vaccination periods

		Children with comorbidity	Children with no comorbidity
Study cohort	Pre *n* = 337	180 (53%)	157 (47%)
	Post *n* = 284	162 (57%)	122 (43%)
Age <2 years	Pre *n* = 204	103 (50.5)	101 (49.5)
	Post *n* = 146	77 (51,4%)	71 (48.6%)
Male gender	Pre *n* = 173	92 (53.7%)	81 (46.3%)
	Post *n* = 152	82 (53.9%)	70 (46.1%)
Severe pneumonia^a^	Pre *n* = 77	52	25
	Post *n* = 87	57	30
PICU care	Pre *n* = 18	15	3
	Post *n* = 27	22	5
Fatal cases	Pre	2	0
	Post	3	0

^a^Significant increase in the post-immunization period (Fishers exact test)

### Characteristics in children with comorbidities

**Fig. 1:**
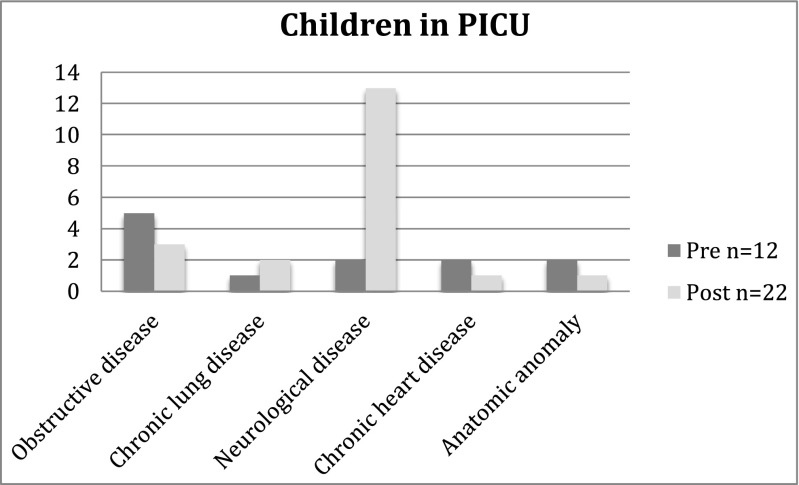
Underlying chronic conditions in children 0–4 years requiring PICU care for pneumonia in pre- and post-period

The majority of children with comorbidity (*n* = 209, 63.1%) had only one underlying disease. However, 91 children (27.5%) had two comorbidities and 31 children (9.4%) had three or more comorbidities. The most common risk factor in both age groups and both periods was obstructive disease requiring regular medication present in approximately half of the patients with comorbidity (Table 1). The effect of immunization on the number of children with comorbidities was most evident in the younger age group. In the post-immunization period, the risk groups including children with chronic lung disease or heart disease, congenital malformations/syndrome and other risk factors decreased in number, but not in proportion of the total number of cases. Among children that required PICU care, the most common risk factors were neurological disease followed by obstructive disease (Fig. 1). Comparing the two periods, an increase in the number of children with neurological disease requiring PICU care was noticed in the post-immunization period.

## Discussion

In this retrospective study, children admitted to hospital for pneumonia have been characterized with respect to comorbidities and severity of disease before and after the introduction of a PCV into the Swedish childhood immunization programme. We conclude that children with underlying comorbidities also have benefitted from the introduction of PCV similar to otherwise healthy children.

Since the introduction of PCV in Sweden, the incidence of pneumonia hospitalizations in children <5 years of age has decreased as previously reported by us and others [10, 1] and is in accordance to several international studies [3, 5, 12]. The study by Berglund et al. covered several parts of Sweden with slightly different vaccine strategies from 2010 and onwards. In our cohort, the largest effect was seen in children <2 years of age, and these young children <2 years had received PCV7 or PCV13 (introduced in Jan. 2010). On the other hand, some children 3–5 years of age in the older age group may not have been offered PCV at all. It is therefore likely that the positive vaccine effect may increase also in the older age group in the coming years when all children have been offered PCV13. Although the trend of a reduced hospitalizations rate is similar in our study compared to Berglund et al., it should be noted that the patient cohorts differed as children with viral pneumonia were excluded in our study.

To understand whether PCV has benefitted children with underlying diseases, we also registered comorbidities associated with CAP hospitalization. Previous studies from the USA [7, 15] suggest that PCV has significantly lower efficacy in preventing IPD in children with some comorbidities such as immunodeficiency, chronic medical conditions and asthma compared with children with no underlying disease. However, our results suggest that children with comorbidities also benefit from the PCV vaccination since the proportion of children with comorbidities and pneumonia was similar before and after the universal introduction of PCV. In addition, it is also important to also stress that the actual number of children with comorbidities decreased in after the introduction of PCV and this was most evident for children with congenital heart disease/malformations/cancer and primary immunodeficiencies. However, one major difference between previous studies and ours is that our study was based on a discharge diagnosis of pneumonia coded as bacterial (J13-J18.9) and not based on culture-verified IPD. Our finding emphasizes the importance of a high vaccine coverage also in children with chronic diseases.

We next sought to elucidate whether the clinical course during hospitalization differed in children with comorbidities compared to otherwise healthy children. Chart review identified a higher proportion of children with comorbidities than the coded discharge diagnosis which emphasize the risk of only using ICD codes. Different routine for coding between institutions as well as the accuracy of the individual paediatrician may impact studies based on discharge diagnosis only. In our cohorts, obstructive disease predominated in the two study periods as also shown in two previous US studies [8, 15]. More than one underlying disease was present in one-third of the children with comorbidity. In children <2 years of age, there were less children with congenital malformations/syndromes or with other diseases (cancer, immunosuppression, diabetes) in the post-immunization period. In the older age group, the distribution of children with comorbidities was rather similar.

Not surprisingly, children with comorbidities in our cohort more often presented with severe pneumonia were more often in need of PICU care. Unfortunately, it was not possible to ascertain from the chart review whether the children with more severe pneumonia actually had received PCV or not, which otherwise would have been interesting. The proportion of children in PICU was much lower in our study compared to recently published data from an active population-based surveillance study of CAP in the USA [8]. Whether this reflects more severe or multiple comorbidities in the different populations or differences in management of severe cases is difficult to ascertain. In the study by Jain et al., 50% had underlying conditions, and 21% of admitted children were in need of ICU care although there may have been a selection bias since one third of possible cases declined participation. The frequency of direct complication to pneumonia (empyema/sepsis) did not change significantly between the study periods in our population contrary to what has been previously shown for empyema in the PCV post-immunization era [6, 9]. Albeit it is possible that our cohort is too small to detect any changes compared to the international studies, the mortality in our selected cohort was low and occurred in children with underlying diseases, which is in accordance with other studies [8, 12].

Several limitations exist with this study. Firstly, a limitation is that the individual vaccination data were not available. However, since data suggests a 98% high vaccine coverage for PCV in children born 2010 in Stockholm county, we think that our estimate of vaccine coverage based on age is reliable [10]. We cannot exclude a selection bias for unvaccinated children with comorbidities, especially for the groups of children with perinatal disease such as BPD or congenital malformations/syndromes since these children may have received their immunizations later in life compared to healthy newborns. However, these children constituted a minor fraction of the children with comorbidities. PCV also has a herd effect in non-vaccinated groups, and this study was designed to evaluate the overall effect of the programme.

Secondly, a limitation is that the initial selection of cases has been made using ICD codes for pneumonia coded as bacterial and we know that there could be children who were given the wrong diagnosis or where comorbidities were not registered. However, to validate discharge diagnosis, we performed chart review in a substantial number of cases. Unfortunately, we could not establish the true aetiology of pneumonia due to incomplete data during chart review, but we know from other studies [8, 17] that all cases were not true ‘bacterial pneumoniaʼ. It was also evident that sampling for microbiological diagnosis differed between the three hospitals included. Within the same time frame as our study, viral PCR diagnostics were introduced in 2007 [20], which may have facilitated and correctly improved the diagnosis of viral pneumonia in children.

In conclusion, we show that all children have benefitted from the reduction of CAP hospitalization after introduction of PCV. In addition, we show that approximately half of all children hospitalized with CAP also have underlying comorbidities. Our data suggests that studies only based on discharge diagnosis may underestimate other important study variables as in our case.

## Abbreviations

CAP: Community-acquired pneumonia
IPD: Invasive pneumococcal disease
LOS: Length-of-stay
NP: Nasopharyngeal
PCR: Polymerase chain reaction
PCV: Pneumococcal conjugate vaccine
PICU: Paediatric intensive care
US: United States of America
